# Genome-wide identification and transcriptional characterization of DNA methyltransferases conferring temperature-sensitive male sterility in wheat

**DOI:** 10.1186/s12864-021-07600-7

**Published:** 2021-04-29

**Authors:** Dan Li, Bian-E Feng, Yong-Jie Liu, Jie Gong, Yi-Miao Tang, Li-Ping Zhang, Bin-Shuang Pang, Ren-Wei Sun, Feng-Ting Zhang, Zhao-Bo Chen, Yong-Bo Wang, Xian-Chao Chen, Ai-Ping Wang, Chang-Ping Zhao, Shi-Qing Gao

**Affiliations:** 1 The Municipal Key Laboratory of the Molecular Genetics of Hybrid Wheat, Beijing Engineering Research Center for Hybrid Wheat, Beijing Academy of Agriculture and Forestry Sciences, Beijing, 100097 China; 2 Shanxi Agricultural University, Taigu, 030800 China

**Keywords:** *Triticum aestivum* L., Temperature-sensitive genic male sterility (TGMS), Stress response, DNA methyltransferases, Transcript abundance

## Abstract

**Background:**

DNA methyltransferase (DMT) genes contribute to plant stress responses and development by de novo establishment and subsequent maintenance of DNA methylation during replication. The photoperiod and/or temperature-sensitive genic male sterile (P/TGMS) lines play an important role in hybrid seed production of wheat. However, only a few studies have reported on the effect of DMT genes on temperature-sensitive male sterility of wheat. Although DMT genes have been investigated in some plant species, the identification and analysis of DMT genes in wheat (*Triticum aestivum* L.) based on genome-wide levels have not been reported.

**Results:**

In this study, a detailed overview of phylogeny of 52 wheat DMT (TaDMT) genes was presented. Homoeolog retention for TaDMT genes was significantly above the average retention rate for whole-wheat genes, indicating the functional importance of many DMT homoeologs. We found that the strikingly high number of TaDMT genes resulted mainly from the significant expansion of the TaDRM subfamily. Intriguingly, all 5 paralogs belonged to the wheat DRM subfamily, and we speculated that tandem duplications might play a crucial role in the TaDRM subfamily expansion. Through the transcriptional analysis of TaDMT genes in a TGMS line BS366 and its hybrids with the other six fertile lines under sterile and fertile conditions, we concluded that *TaCMT-D2*, *TaMET1-B1*, and *TaDRM-U6* might be involved in male sterility in BS366. Furthermore, a correlation analysis showed that *TaMET1-B1* might negatively regulate the expression of *TaRAFTIN1A*, an important gene for pollen development, so we speculated regarding an epigenetic regulatory mechanism underlying the male sterility of BS366 via the interaction between *TaMET1-B1* and *TaRAFTIN1A*.

**Conclusions:**

Our findings presented a detailed phylogenic overview of the DMT genes and could provide novel insights into the effects of DMT genes on TGMS wheat.

**Supplementary Information:**

The online version contains supplementary material available at 10.1186/s12864-021-07600-7.

## Background

DNA methylation is a heritable epigenetic modification widely found in bacteria, animals and plants and is instrumental in transposable element (TE) suppression, gene silencing, genomic imprinting and chromosome inactivation [1, 2]. DNA methylation is catalyzed by DNA methyltransferase (DMT) that introduces a methyl group at the C5 or N4 position of cytosine or the N6 position of adenine. C5-Cytosine DNA methylation (simply named here as DNA methylation) is the most prevalent among all these modifications, especially in plants [3, 4]. DNA methylation occurs in multiple sequence contexts, including CG and CHG, both of which are symmetric, and CHH, which is asymmetric (H = A, T, or C) [5, 6]. Two major methods of modulating DNA methylation have been found, including de novo methylation and maintenance methylation [7, 8]. Maintenance methylation occurs during DNA replication in hemimethylated CG and CHG contexts. De novo methylation occurs at all three unmethylated cytosines [3, 9].

In plants, DNA methylation is controlled by four subfamilies of DMT genes: methyltransferase 1 (MET1, the homologs of mammalian DNMT1), chromomethylase (CMT, plant-specific), domains rearranged methyltransferase (DRM, the homologs of mammalian DNMT3) and DNA methyltransferase 2 (DNMT2) [10–12]. MET1 genes played a pivotal role in maintaining methylation in the CG context. *Arabidopsis thaliana* harbored four MET1 homologs, but only the loss-of-function of *MET1* bore phenotypic consequences. Ablation of *MET1* resulted in nearly complete loss of CG methylation in *Arabidopsis thaliana* [13–15]. In rice (*Oryza sativa* L.), there were two MET1 genes (*OsMET1A* and *OsMET1B*), but only *OsMET1B* (*OsMET1–2*) plays an essential part in maintaining CG methylation during normal growth and development. A null mutation of *OsMET1B* led to genome-wide hypomethylation [16]; however, knockout of *OsMET1A* (*OsMET1–1*) did not show alteration of DNA methylation at assessed marker loci [17]. The maintenance of major CHG methylation is accomplished by CMT3 [18]. There were three CMT genes (*AtCMT1*, *AtCMT2,* and *AtCMT3*) in *Arabidopsis thaliana*, and *AtCMT3* is the main CHG methyltransferase. In a mutant of *AtCMT3*, strong depletion of CHG methylation was observed [19]. In addition, previous research showed that CMT2 could partly partake in the maintenance of CHH methylation via cross-talk with histone modifications in plants [11, 12]. In all three contexts, DRMs are primarily responsible for catalyzing de novo DNA methylation, which is achieved by the RNA-directed DNA methylation (RdDM) pathway in plants. RdDM involves two specific RNA polymerases, RNA Pol IV and Pol V, as well as 24 nt small interfering RNAs (siRNAs) [20–23]. DRM2 is required for de novo DNA methylation of all three contexts and for the maintenance of non-CG methylation [24, 25]. After the targeted destruction of *OsDRM2*, the CG and non-CG methylation levels were reduced, and de novo methylation mediated by the RdDM process was deficient [26]. Based on sequence homology, DNMT2 was originally considered a DNA methyltransferase. However, robust DNA methyltransferase activity could not be observed using DNMT2 preparations, and the considerable variability in the target DNA sequences showed that DNMT2 enzymes were actually tRNA transferases [27–29].

Comprehensive studies have showed that plant DNA methylation was predominantly associated with growth and development [30, 31], secondary metabolism [32, 33] and various stress responses, including salt stress [34, 35], drought stress [36, 37], and cold stress [38, 39]. Since DNA methylation is involved in regulating a wide range of biological processes, DMT genes have been identified and analyzed in several plant species, such as *Arabidopsis thaliana* [40, 41], rice [42, 43], maize [44, 45], and wheat [46]. However, to the best of our knowledge, no investigator has focused on the identification and analysis of DMT genes in wheat at the whole-genome level.

Wheat (*Triticum aestivum* L.) is one of the most important staple crops worldwide, contributing a significant amount of proteins and calories to the global human population [47, 48]. Given a growing world population coupled with increasingly challenging cultivation conditions, facilitating breeding and large-scale adoption of hybrid wheat are of great importance to the food supply [49, 50]. The two-line system based on photoperiod and/or temperature-sensitive genic male sterile (P/TGMS) lines plays an important role in hybrid seed production of wheat [51]. The male sterility of the P/TGMS line is heritable but regulated by an appropriate temperature or photoperiod. In the TGMS line of wheat, pollen viability is dramatically decreased to complete sterility at a certain low temperature occurring during reproductive development [52, 53]. Temperature stress may lead to complete sterility and severe losses in grain yield during reproductive development mainly because the reproductive stage is especially sensitive to environmental stresses [54]. Specifically, male reproductive organs are known to be more sensitive to temperature stress than female organs in higher plants [55]. Plants have developed diverse mechanisms to survive extreme environmental conditions. Changes in DNA methylation have been shown to play a crucial part in plant stress responses [6]. Additionally, some recent reports have revealed that DNA methylation levels are affected by DMTs and regulate the expression of genes involved in cytoplasmic male sterility in rice, maize, and wheat [56–58]. However, few studies have been reported that explore the roles of DMTs in TGMS wheat during the reproductive stage.

To better understand the dynamic evolution of the DMT gene in wheat and to facilitate further research on this important gene family, all members of DMT genes in wheat were identified, and their phylogenetic relationships and gene structures were investigated. We found that the wheat DRM subfamily was significantly larger than expected and hypothesize that high homoeolog retention in the DRM subfamily contributed to the expansion of wheat DMT genes. Moreover, the transcript abundance of DMT genes under sterile and fertile conditions was analyzed. Notably, we found that most TaDMT genes were significantly upregulated under sterile conditions compared to fertile conditions. After further expressional analysis, we concluded that *TaCMT-D2*, *TaMET1-B1*, and *TaDRM-U6* were more likely associated with male sterility in the TGMS line BS366, particularly the interaction between *TaMET1-B1* and *TaRAFTIN1A*. The results of this study present a detailed overview of the phylogeny of DMT genes and provide novel insights into the functional roles of DMT genes in the male sterility of TGMS wheat.

## Results

### Identification and phylogenetic analysis of DMT genes

**Fig. 1:**
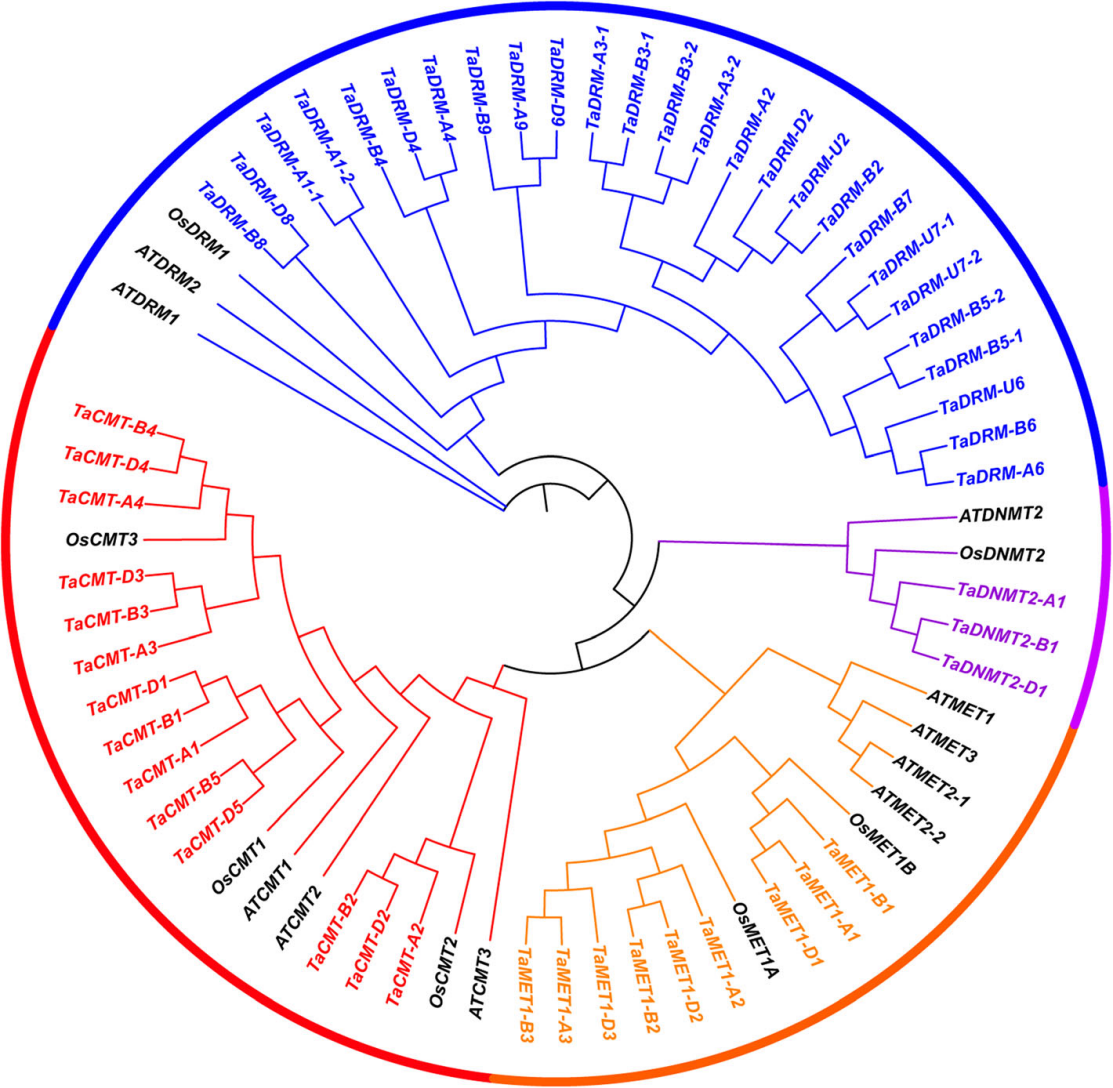
Phylogenetic analysis of DMT proteins from *Arabidopsis*, rice and wheat. A maximum likelihood phylogeny of DMT domain proteins from *Arabidopsis*, rice, and wheat was generated using MEGA-X [60]. TaDMT genes are colored according to subclade, whereas AtDMT genes and OsDMT genes are in black

To analyze the function of DMT genes, we performed genome-wide identification and phylogenetic analysis of DMT genes in wheat. A total of 52 DMT genes were identified on the basis of the functional annotation (Pfam domains) in the IWGSC archive v.1.0 [59]. We named all TaDMT genes according to their subfamily association and subgenome location (Supplemental Table S1). *Arabidopsis* and rice had 10 and 7 DMTs, respectively (Fig. 1). Wheat had the largest number of DMTs across the three species; this was partly the result of the hexaploid nature of wheat. However, even after correcting for ploidy level, the number of DMT genes in wheat was significantly higher than that in rice (c. 2.5-fold higher; χ^2^ test, *P* = 0.02). The increase in the number of TaDMT genes was mainly a result of the gene counts in the TaDRM subfamily, which was significantly larger than expected (χ^2^ test, *P* = 0.01). The numbers of genes in the remaining subfamilies were not significantly different from the expected 3:1 ratio.

To elucidate the phylogenetic relationship of DMT genes from *Arabidopsis thaliana*, rice (*Oryza sativa* L.), and wheat, a total of 69 DMT protein sequences were used. A maximum likelihood phylogenetic tree of DMT genes showed that TaDMT genes could be naturally grouped into four subfamilies: MET1, CMT, DRM, and DNMT2 (Fig. 1). In all subclades, the DMT phylogeny roughly followed species phylogeny. As the *Arabidopsis* genes were a sister group related to the grass genes, OsDMT genes were more closely related to a triad of wheat homologs such as those in the MET1 and CMT subfamilies (Fig. 1).

### Chromosomal location and homoeologous genes of DMT genes in the wheat genome

**Fig. 2:**
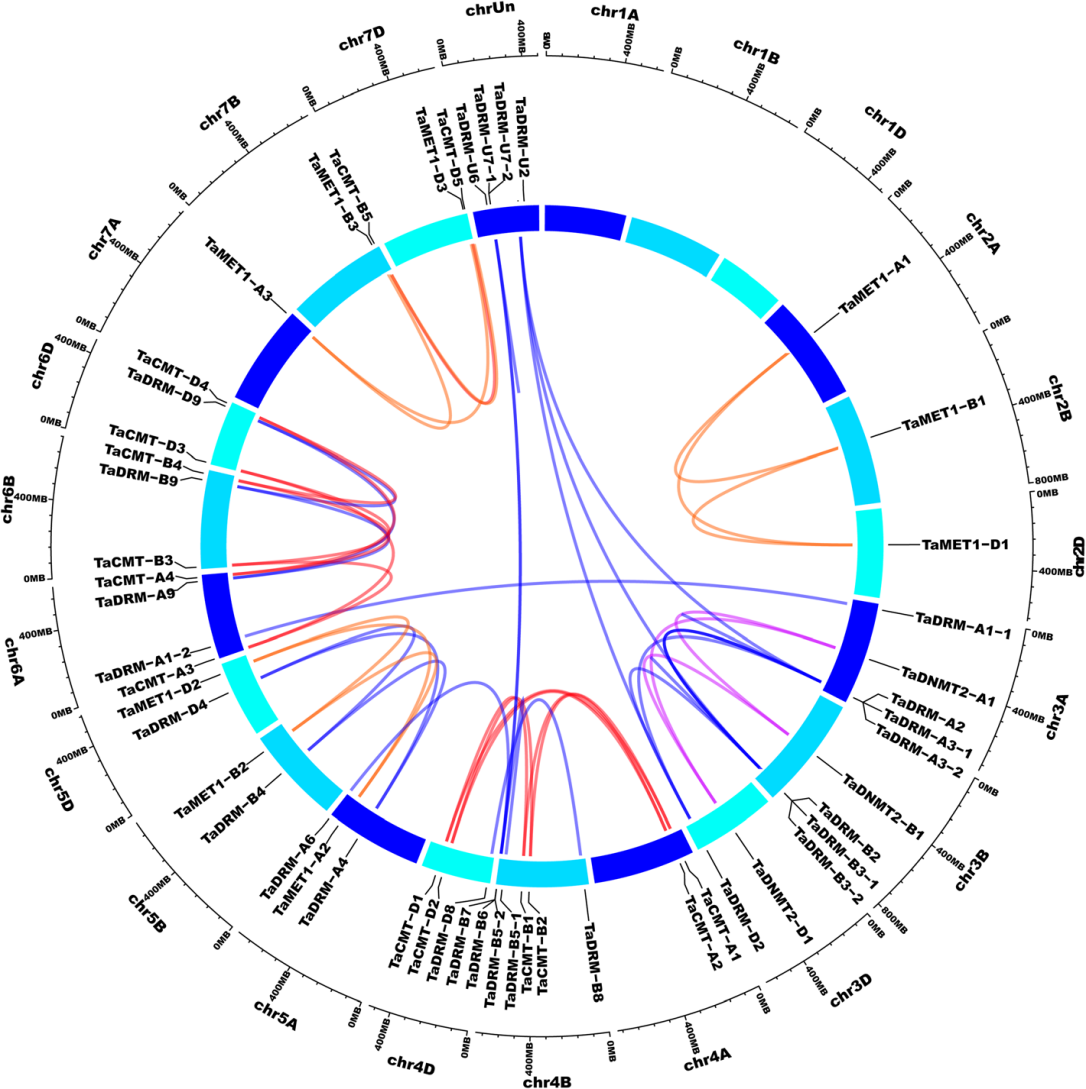
Chromosomal location and paralogs of DMT genes in the wheat genome. All TaDMT genes were mapped to their respective locus in the wheat genome in a circular diagram. Subgenomes are indicated by various shades of blue. Homeologous genes were inferred by phylogeny and linked with subfamily-specific colors as in Fig. 1 (inside the circle)

DMT genes were unevenly distributed across wheat chromosomes: chr1A, chr1B, and chr1D had no TaDMT gene, and the highest number of genes was located on chr4B (7 genes) (Fig. 2). Interestingly, different members of DMT gene subfamilies were located in chromosomal regions that might represent homoeologous segments resulting from ancestral events [49]. Approximately 69% (12 triads) of the 52 DMT genes identified were present in triads. Additionally, 23% (six pairs) of DMT genes lost one homoeologous gene of the triads in the wheat genome. Thus, the high homoeologous retention rate can partly explain the high number of wheat DMT genes. Intriguingly, there were 5 paralogs in the wheat genome that were all from the DRM subfamily, indicating that tandem duplication might an important mechanism for subfamily expansion (Fig. 2).

### Conserved motifs and gene structures of DMTs in wheat

**Fig. 3:**
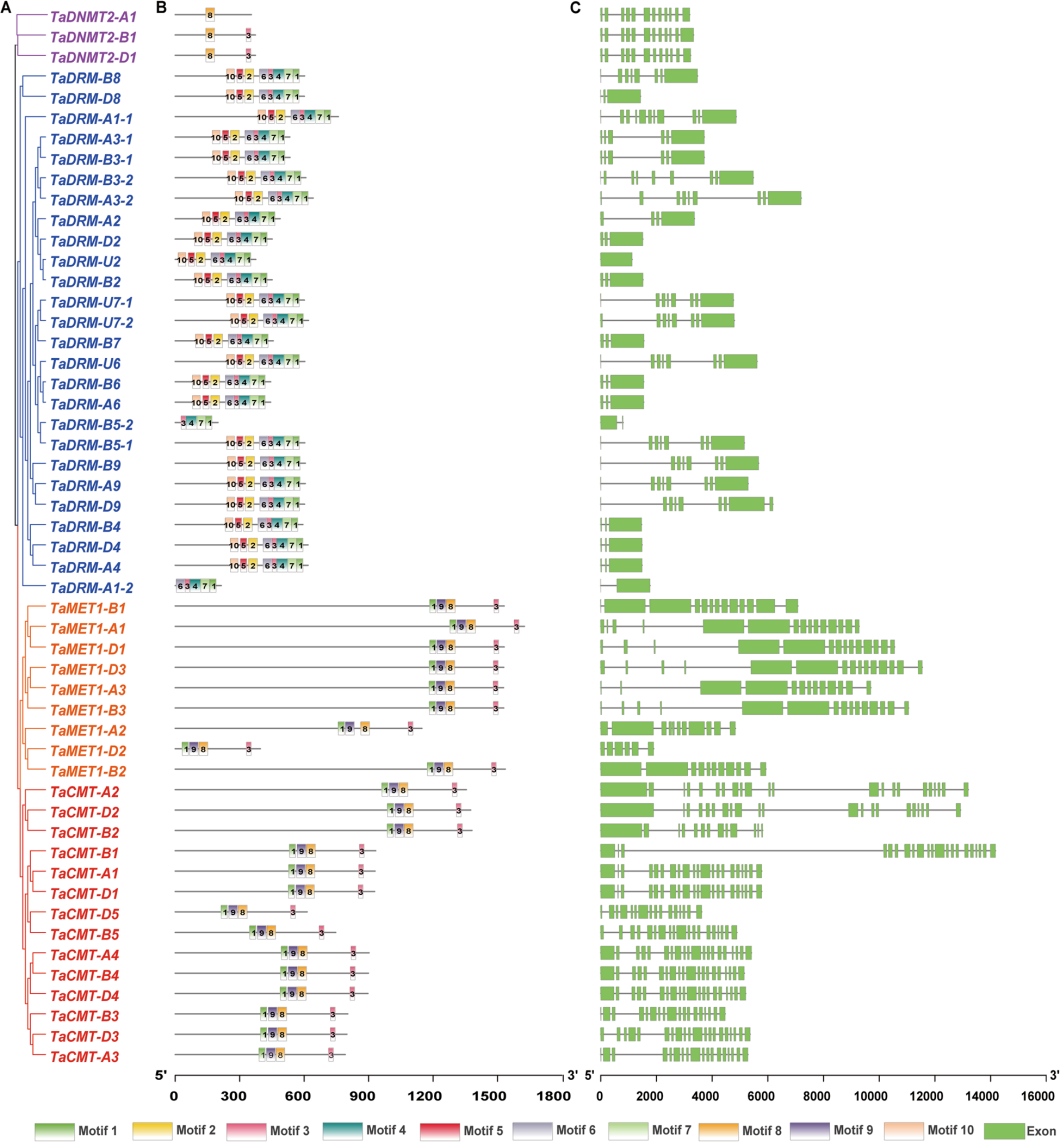
Phylogenetic relationships (**a**), conserved motifs (**b**), and gene structures (**c**) of DMT genes in wheat. TaDMT genes are colored according to subclade as in Fig. 1

Phylogenetic relationships of wheat DMTs showed that the MET1 and CMT subfamilies were more similar and belonged to the same clade (Fig. 3a). Furthermore, the motifs in all members of TaDMT genes were analyzed. A total of 10 different motifs were identified in 52 TaDMT proteins (Fig. 3b). Among the motifs, motifs 1, 3, 8, and 9 were found in all TaCMT and TaMET1 proteins, hence, these two subfamily proteins were clustered together in a small branch. TaDNMT2 was the smallest subfamily in the wheat phylogenetic tree and contained only 3 genes. The TaDNMT2 proteins included motifs 3 and 8. Most TaDRM genes were composed of 8 motifs, including in sequential order motifs 10, 5, 2, 6, 3, 4, 7, and 1, which were significantly different from the structure of the other three TaDMT genes. This finding was consistent with the results of the gene structures of TaDMT genes (Fig. 3c). The TaDMT genes exhibited different exon-intron organizational patterns. TaCMT genes had the largest number of exons; however, TaDRM genes had the smallest number of exons.

### DMT genes were significantly enriched in the thermosensitive genic male sterile line BS366 under sterile and fertile conditions

**Fig. 4:**
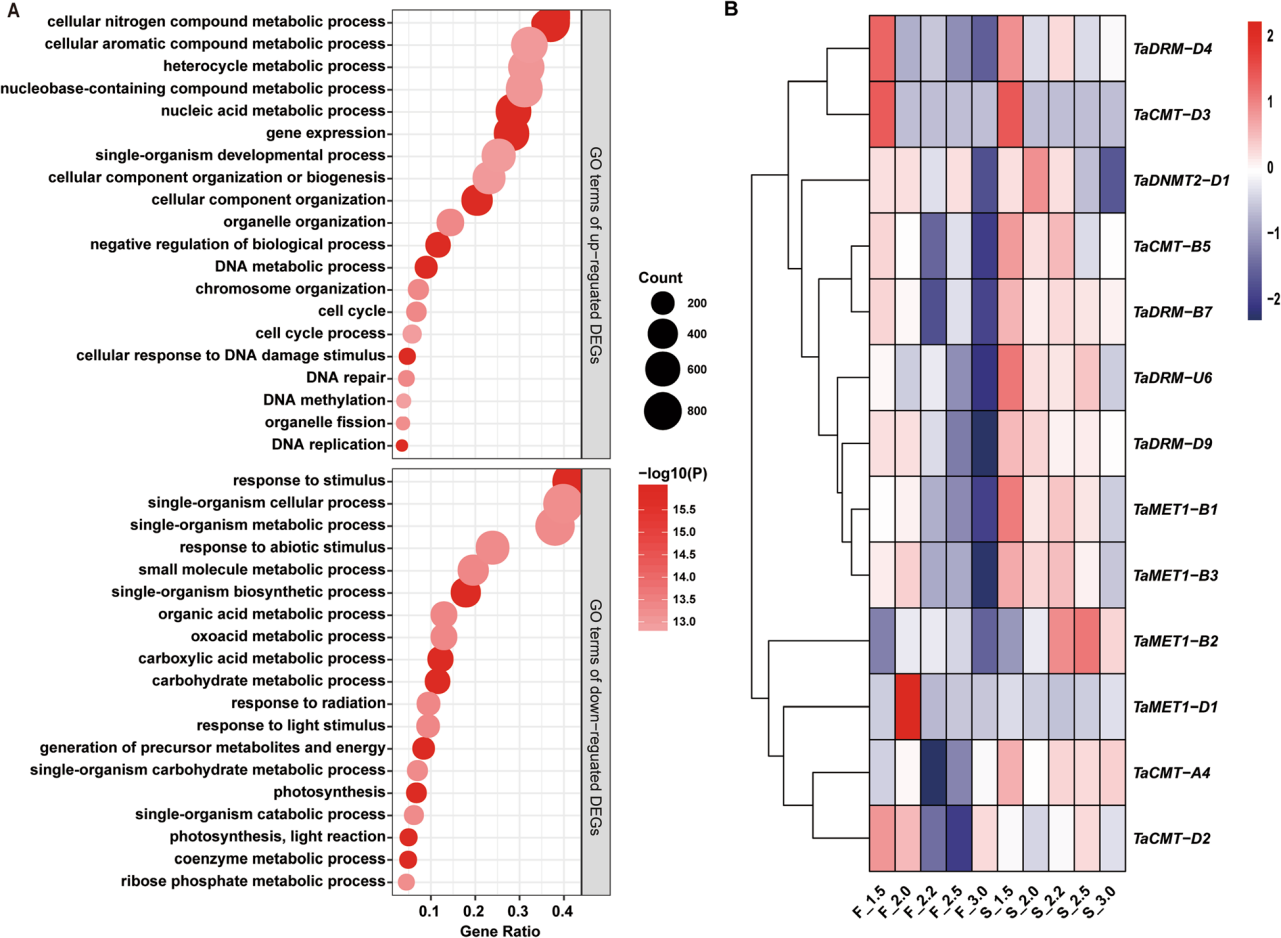
Enriched analysis of differentially expressed genes and expressed transcripts of TaDMT genes under sterile and fertile conditions in BS366 based on the microarray data. **a** The enriched biological process GO terms using differentially expressed genes in BS366. Significant enrichment is indicated by -log10 (*P* value). The above box indicates GO terms of upregulated expressed genes; the box below indicates GO terms of downregulated expressed genes. **b** The hierarchical clustering of expressed TaDMT genes according to their relative transcript levels in 10 samples. Four main clusters are indicated with colors. The letter “S” indicates sterile treatment; “F” indicates fertile treatment. The numbers at the bottom of each panel (1.5, 2.0, 2.2, 2.5, and 3.0) represent the lengths of different anther samples

A published GeneChip® data set was used to seek differently expressed genes (DEGs) under sterile and fertile conditions in the temperature-sensitive genic male sterile line BS366 anthers during meiosis [52]. The up and downregulated probe sets were used for enrichment analysis by Gene Ontology (GO). The metabolic process and DNA methylation were the major overrepresented GO terms in the biological process (Fig. 4a). DNA methylation, the most common epigenetic modification, is an important link between phenotypes and genotypes, so we speculated that it might regulate metabolic process or other processes which eventually modulate male fertility in BS366. To further understand the function of DNA methylation, we first analyzed the function of DMT genes in wheat and the expressed transcripts of DMT genes in BS366.

To clarify the potential roles of TaDMT genes involved in male sterility, we analyzed the transcript abundance of TaDMT genes in BS366 based on the GeneChip® data. In this study, there were 13 TaDMT genes expressed, and 10 of them were differentially expressed under sterile and fertile conditions (Fig. 4b). The 9 TaDMT genes were upregulated, and only *TaMET1-D1* was downregulated under sterile conditions compared to fertile conditions. *TaMET1-B1* and *TaCMT-D2* were significantly differentially expressed in the 1.5-mm anther stage. *TaMET1-D1* was significantly differentially expressed in the 2.0-mm anther stage. *TaMET1-B1*, *TaCMT-A4*, *TaDRM-B7*, *TaMET1-B*2, *TaMET1-B3*, *TaCMT-B5*, and *TaCMT-D2* were significantly differentially expressed in the 2.2-mm anther stage. *TaMET1-B1*, *TaMET1-B2*, *TaCMT-D2*, *TaDRM-D9*, and *TaDRM-U6* were significantly differentially expressed in the 2.5-mm anther stage. *TaMET1-B1*, *TaDRM-B7*, *TaMET1-B2*, *TaMET1-B3*, *TaCMT-B5*, *TaDRM-D9*, and *TaDRM-U6* were specifically differentially expressed in the 3.0-mm anther stage. Almost all differential expression of TaDMT genes occurred during the 2.2-, 2.5-, or 3.0-mm anther stage. From the 2.2-mm anther stage, mitosis ceased and transited to meiosis I according to morphological observations. The result reinforced the conclusion that cold stress contributed to aberrant cytokinesis during male meiosis I in BS366 [52]. Therefore, we speculated that the 2.2-mm, 2.5-mm, and 3.0-mm periods were the key periods affecting male sterility in BS366. Therefore, we chose *TaMET1-B1*, *TaCMT-A4*, *TaDRM-B7*, *TaMET1-B2*, *TaMET1-B3*, *TaCMT-B5*, *TaCMT-D2*, *TaDRM-D9*, and *TaDRM-U6* to further assess their expression.

### Transcript abundance of TaDMT genes in fertile lines and hybrids of wheat under sterile and fertile conditions

**Fig. 5:**
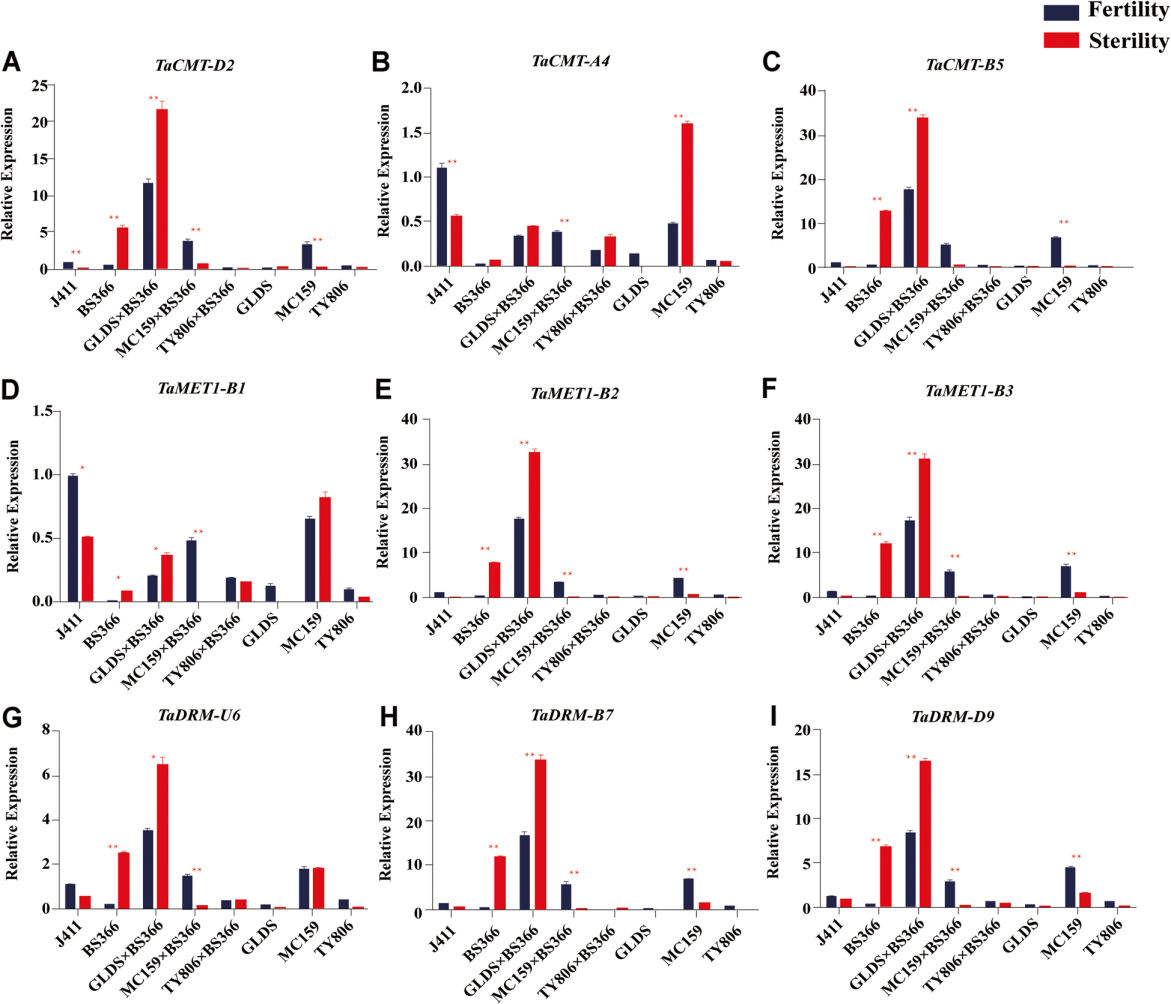
Transcript abundance of TaDMT genes under sterile and fertile conditions in wheat. Expression profiles of the TaDMT genes in BS366, four fertile wheat lines and three hybrids by qRT-PCR; BS366 is a sterile line; J411, GLDS, MC159, and TY806 are fertile lines; GLDS×BS366, MC159 × BS366, and TY806 × BS366 are three different fertility-restored hybrids. Data are presented as the mean ± standard deviation (SD). All experiments were repeated at least three times. ** indicates *P*-value < 0.01, * indicates P-value < 0.05 (Stertility vs. Fertility, Student’s t test)

To verify the expression of the 9 TaDMT genes involved in male fertility in BS366, the anthers sampled from BS366, four fertile lines (J411, TY806, MC159, and GLDS) and three hybrids (GLDS×BS366, MC159 × BS366, and TY806 × BS366) at the meiosis stage were used. *TaCMT-D2*, *TaCMT-B5*, *TaMET1-B1*, *TaMET1-B2*, *TaMET1-B3*, *TaDRM-U6*, *TaDRM-B7*, and *TaDRM-D9* were variously upregulated in BS366 under sterile compared to fertile conditions, but they were downregulated or not differently expressed in the four fertile lines (Fig. 5). *TaCMT-A4* gene was not differentially expressed in BS366 while it was differentially expressed in J411 and MC159 under sterile compared to fertile conditions (Fig. 5b). BS366 and three fertile lines (TY806, MC159, and GLDS) have been used to generate three different fertility-restored hybrids: TY806 × BS366 (91.17%), MC159 × BS366 (90.60%), and GLDS×BS366 (89.83%) [61]. Therefore, TY806, MC159, GLDS, and their hybrids with BS366 were considered excellent materials to assess the potential association of TaDMT genes with male sterility in BS366. Upregulated expression was detected for *TaCMT-D2*, *TaCMT-B5*, *TaMET1-B1*, *TaMET1-B2*, *TaMET1-B3*, *TaDRM-U6*, *TaDRM-B7*, and *TaDRM-D9* in GLDS×BS366, while their expression were downregulated or not different in TY806 × BS366 and MC159 × BS366 under fertile and sterile conditions (Fig. 5). Bases on the above results, we speculated that *TaCMT-D2*, *TaCMT-B5*, *TaMET1-B1*, *TaMET1-B2*, *TaMET1-B3*, *TaDRM-U6*, *TaDRM-B7* and *TaDRM-D9* might be associated with male sterility in BS366.

**Fig. 6:**
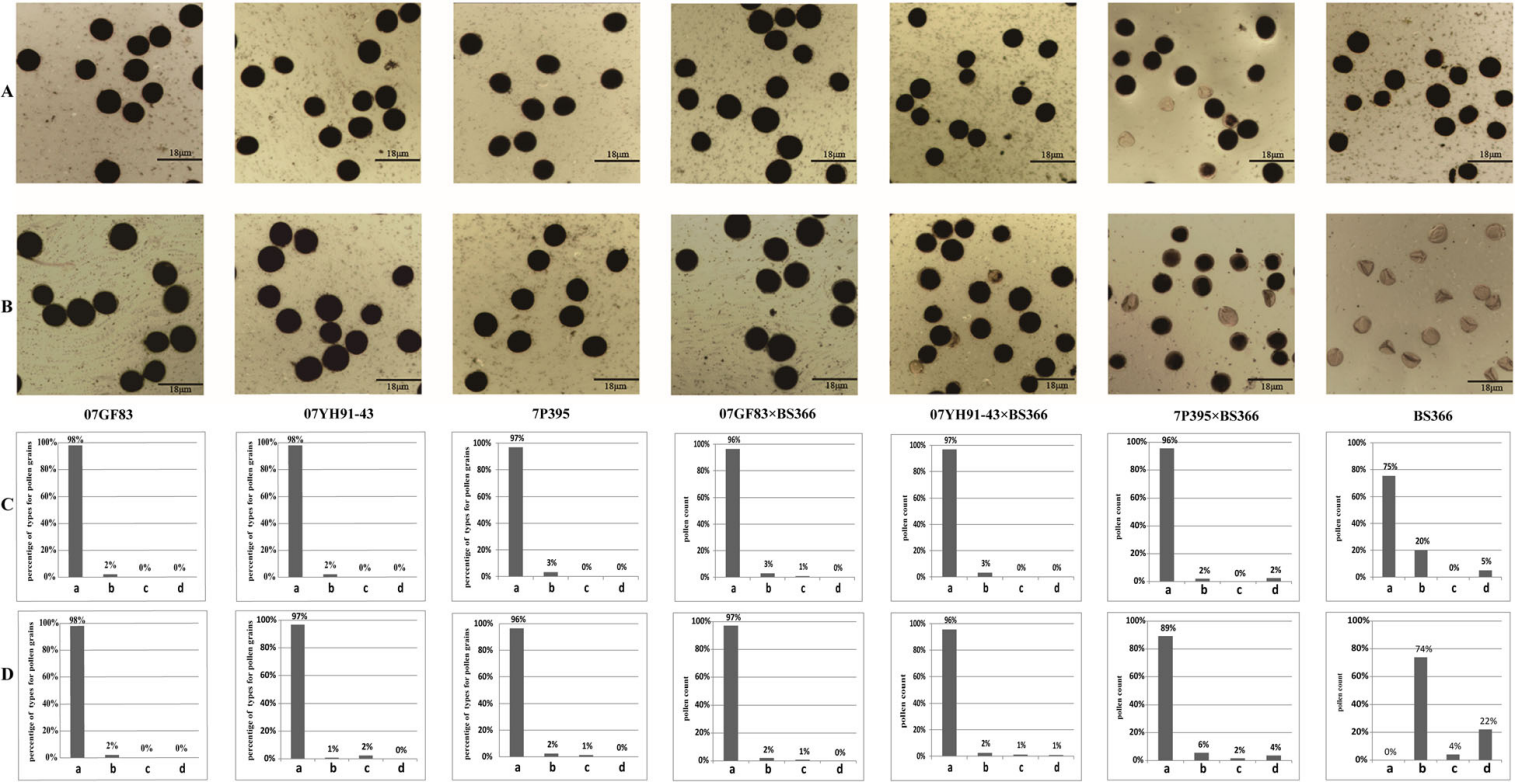
Iodine staining of pollen grains and statistical analysis in different wheat lines. The wheat materials were taken separately from fertile (**a**, **c**) and sterile (**b**, **d**) conditions. **a** and **b** showed the iodine staining data. *Scale bar = 18* μm; **c** and **d** showed the statistical analysis data. The total pollen counts used were over 240 in every line. a-d on the x-axis represent four different shape-color combinations or types for the iodine-stained pollen grains: **a** circular, opaque, and dark brown/black; **b** circular, opaque, or partially transparent and light brown/black; **c** circular, transparent, and light yellow; and **d** transparent with an irregular shape and light yellow

**Fig. 7:**
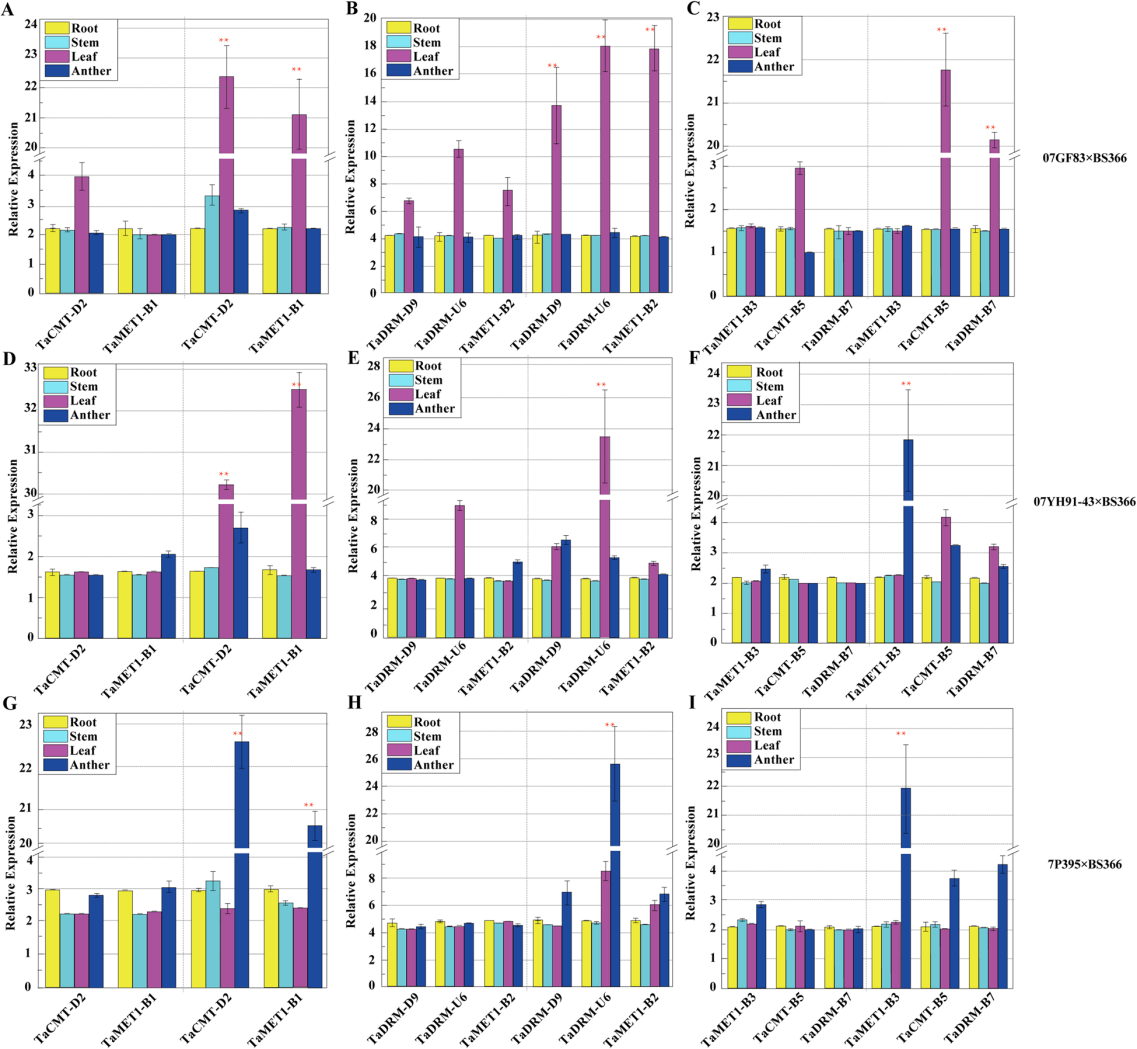
Tissue-specific expression profiles of TaDMT genes in different hybrids under fertile and sterile conditions. **a-i** show the expression levels of eight TaDMT genes under fertile (left) and sterile (right) conditions. 07GF83 × BS366, 07YH91–43 × BS366, and 7P395 × BS366 are high-, medium-, and low- fertility F_1_ hybrids. Data are presented as the mean ± standard deviation (SD). All experiments were repeated at least three times. ** indicates P-value < 0.01 (Stertility vs. Fertility, Student’s t test)

To validate our speculation, we further analyzed the tissue-specific expression patterns of the eight TaDMT genes in roots, stems, leaves, and anthers using another three different fertility-restored F_1_ hybrids 07GF83 × BS366 (97%), 07YH91–43 × BS366 (96%), and 7P395 × BS366 (89%) under sterile conditions (Fig. 6b and d). These hybrids were crossed by high-(07GF83), medium-(07YH91–43), and low-restorer lines (7P395) with BS366. Likewise, they were also prominent materials to evaluate the underlying association of TaDMT genes with male sterility in BS366. In 07GF83 × BS366, the expression levels of *TaCMT-D2*, *TaCMT-B5*, *TaMET1-B1*, *TaMET1-B2*, *TaDRM-U6*, *TaDRM-B7*, and *TaDRM-D9* in leaves were significantly higher under sterile conditions than that under fertile conditions (Fig. 7a-c). In 07YH91–43 × BS366, *TaCMT-D2*, *TaMET1-B1*, and *TaDRM-U6* expression were significantly higher in leaves under sterile conditions than that under fertile conditions; *TaMET1-B3* expression was significantly higher in anthers under sterile conditions than that under fertile conditions (Fig. 7d-f). In 7P395 × BS366, *TaCMT-D2*, *TaMET1-B1*, *TaMET1-B3*, and *TaDRM-U6* expression was significantly higher in anthers under sterile conditions than that under fertile conditions (Fig. 7g-h). Considering the differing fertility of 07GF83 × BS366, 07YH91–43 × BS366, and 7P395 × BS366, *TaCMT-D2*, *TaMET1-B1* and *TaDRM-U6* displayed tissue-specific differential expression in anthers in the low fertility line 7P395 × BS366. Therefore, we concluded that *TaCMT-D2*, *TaMET1-B1*, and *TaDRM-U6* might take part in male sterility in BS366.

### Expressional correlation analysis among candidate TaDMT genes and the other DEGs in BS366 under fertile and sterile conditions

**Fig. 8:**
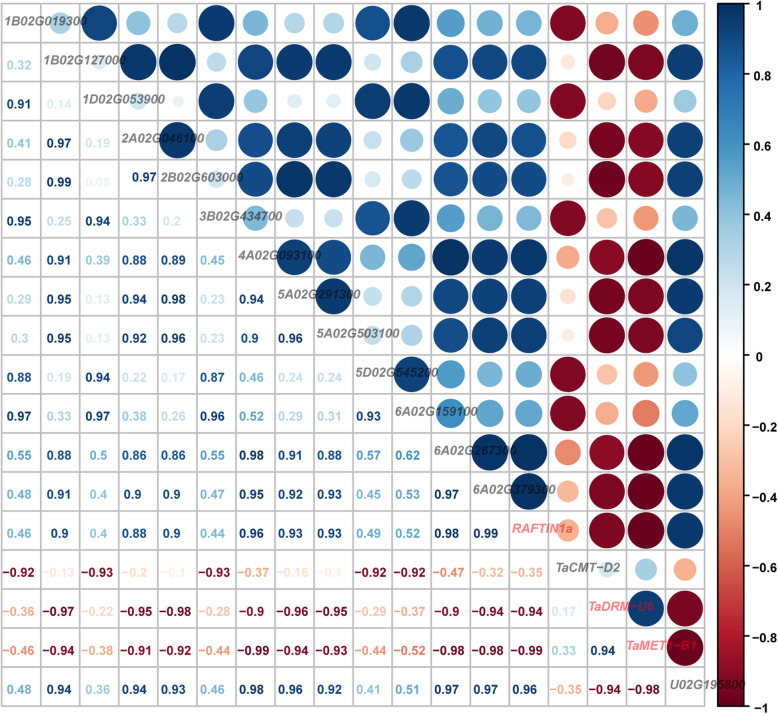
Expressional correlation analysis among candidate TaDMT genes and DEGs in BS366 under fertile and sterile conditions. The plots on the diagonal show the names of each gene. The values below the diagonal are Pearson correlation coefficients between genes, and the plots above the diagonal are circle plots of compared genes. The size of the circle represents the degree of correlation. Red values and circles indicate a negative correlation; blue values and circles indicate a positive correlation

To search genes that might interact with the candidate TaDMT genes, correlation analyses of expressional levels among the DEGs in BS366 under fertile and sterile conditions were conducted. The degree of correlation varied substantially among the genes, and Fig. 8 showed the highly negatively correlated genes with the candidate TaDMT genes. *TaRAFTIN1A*, an important gene for pollen development [62], and *TraesCS4A02G093100* were the most highly negatively correlated with *TaMET1-B1* (r = − 0.99, Fig. 8). We assumed that the interaction between *TaMET1-B1* and *TaRAFTIN1A* might play an important role in the male sterility of BS366. In addition, *TaRAFTIN1A* has a relatively high negative correlation with *TaDRM-U6* (r = − 0.94, Fig. 8)*.* Moreover, we analyzed the *cis*-acting elements in the promoter sequences of *TaCMT-D2*, *TaMET1-B1*, *TaDRM-U6*, and *TaRAFTIN1A* (Supplemental Fig. S3). According to the prediction of *cis*-acting elements, *TaCMT-D2*, *TaMET1-B1*, and *TaRAFTIN1A* genes contained the LTR element that was considered in response to low temperature.

### The expression and methylation levels of *TaRAFTIN1A* in wheat under sterile and fertile conditions

**Fig. 9:**
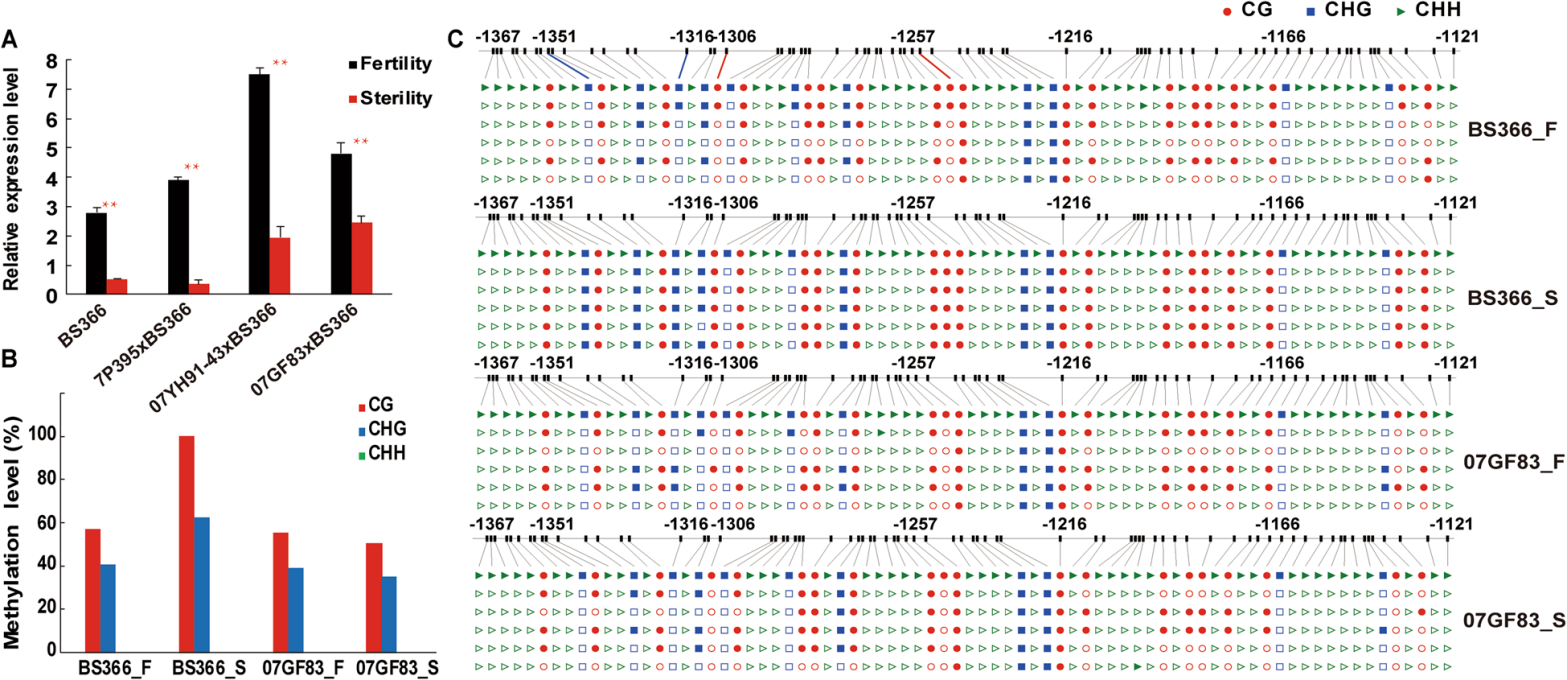
The transcript and methylation level of *TaRAFTIN1A* in BS366 under sterile and fertile conditions. **a** The expression level of *TaRAFTIN1A* in BS366 and three hybrids under sterile and fertile conditions. Data were presented as the mean ± standard deviation. All experiments were repeated at least three times. ** indicates P-value < 0.01 (Stertility vs. Fertility, Student’s t test). **b** The methylation level of *TaRAFTIN1A* promotor in BS366 and a fertile wheat line 07GF83 under sterile and fertile conditions **c** Bisulfite sequencing in the *TaRAFTIN1A* promotor sequence The red, blue and green columns in the histograms refer to the collective methylation levels (in percentage) of CG, CHG, and CHH, respectively

To verify the relationship between *TaMET1-B1* and *TaRAFTIN1A*, the transcript and DNA methylation levels of *TaRAFTIN1A* was further examined. The expression levels of *TaRAFTIN1A* in the anthers of BS366 and its three hybrids (07GF83 × BS366, 07YH91–43 × BS366, and 7P395 × BS366) were significantly downregulated under sterile conditions than that under fertile conditions (Fig. 9a). After CpG island prediction, the *TaRAFTIN1A* sequence from − 1121 to − 1367 was selected to be applied with bisulfite sequencing. We identified three different DNA methylation patterns (CG, CHG, and CHH) in BS366 and 07GF83 under fertile and sterile conditions. Because very few loci of CHH type were detected, we focused on CG and CHG types in the subsequent analysis. We found that the average CG and CHG methylation levels in BS366 were with a difference by more than 20% under sterile conditions than that under fertile conditions (Fig. 9b), but not in the fertile line 07GF83. There were significant differences among four CpG sites (− 1351, − 1316,-1306, and − 1257) with low methylation in BS366 under fertile conditions as compared with that under sterile conditions (Fig. 9c). From these results, we speculated that the expression level of *TaMET1-B1* might be negatively correlated with *TaRAFTIN1A* expression and positively correlated with the methylation levels of the CpG islands in promoter regions.

## Discussion

### Structural and evolutionary features of DMT genes in wheat

DMTs play an important role in modulating DNA methylation and gene expressional levels, which are involved in various stress responses [6]. DMTs are, therefore, promising targets for crop breeding and improvement. In this study, a total of 52 DMT genes were identified in wheat (Supplemental Table S1; Fig. 1). The number of DMT genes discovered in wheat was higher than that in *Arabidopsis* (10) and rice (7). The number of DMT genes was ~ 7.4 times higher in wheat than in rice. Leaving hexaploidy aside, the number of DMT genes in wheat was significantly higher than that in rice (c. 2.5-fold higher; χ2 test, *P* = 0.02). Further, we found that the strikingly high number of TaDMT genes was mainly a result of the significant expansion of genes in the TaDRM subfamily (χ2 test, *P* = 0.01). DNA methylation has long been thought to mostly control repetitive elements within genomes [63]. The insertion and deletion of repetitive elements such as transposable elements (TEs) strongly impact genome size and evolution [64]. TEs may spread rapidly, resulting in deleterious or null mutations within genomes, which present a threat to genome stability and integrity, resulting in plant structural and developmental defects [65]. To prevent this threat, DNA methylation mechanisms can be triggered by various mechanisms [66]. DNA methylation was considered one of the key components in understanding divergence in genome evolution among plants [67]. The expansion of wheat DMT genes might contribute to the large genome size of wheat. In all, 69% (12 triads) of wheat DMT genes could be assigned to 1:1:1 homoeologous groups (Fig. 2). This is significantly above the average homoeologous retention rate (35.8%) in wheat [49]. Thus, the high homoeologous retention rate can partly explain the high number of wheat DMT genes. In addition, there were 5 paralogs in the wheat genome that were all from the DRM subfamily. Hence, we deduced that tandem duplication and a high homoeologous retention rate may be the key factors in the evolution of DMT genes in wheat.

In phylogenetic classification, DMT genes were assigned to 4 conserved subfamilies, namely, MET1, CMT, DRM, and DNMT2. Except for the DRM subfamily, the numbers of genes in the remaining subfamilies were not significantly different from the expected 3:1 ratio after factoring in the ploidy level. In all subclades, the DMT phylogeny roughly followed species phylogeny, and OsDMT genes were more closely related to the triad of wheat homologs. DMT genes diverged prior to the split of monocots and dicots, so we speculated that the functions of DMT genes may be different between dicots and monocots.

Although the number of DMT genes varied widely among different plant species, we found that they had conserved domains (Supplemental Fig. S1). All members of the DMT genes identified in this study contained the DNA-methylase domain, indicating that this domain may be the core domain of DMT genes. Some studies have shown that the absence of the DNA-methylase domain means that the DMT proteins unable to normally methylate cytosine [6, 68]. All members of TaDRM (27) and TaDNMT2 (3) contained only the DNA-methylase domain. The BAH domain (bromo-adjacent homology) existed in almost all the members of TaMET1 and TaCMT. MET1 and CMT3 mainly maintain CG and CHG methylation, respectively [5], so we speculated that the BAH domain plays a key role in the maintenance of symmetrical methylation. Indeed, when the BAH domain was disrupted in *AtCMT1*, the mutated AtCMT1 protein was reported to function abnormally in methylation maintenance in *Arabidopsis* [69]. Similarly, with ablation of the BAH domains within the DNMT1 (MET1) locus, the internally deleted protein lost the ability to mediate maintenance methylation during S phase in embryonic stem (ES) cells [70]. Furthermore, almost all the members of TaMET1 and TaCMT also contained extra chromo and DNMT1-RFD domains, respectively. We speculate that the chromo and DNMT1-RFD domains are responsible for the recognition of hemi-methylated CHG and CG dinucleotides, respectively. Among the motifs, motif 3 (Fig. 3b) was found in all the TaDMT protein except for TaDNMT2-A1. We speculated that the sequence in motif 3 might be the core sequence of the DNA-methylase domain (Fig. S2). All TaCMT and TaMET1 proteins had sequential order motifs 1, 9, 8, and 3, so these two subfamily proteins were clustered together in a small branch. Sequence analysis for conserved domains/motifs of DMT genes shed light on structural and functional conservation and/or unique features of the wheat DMT homologs.

### DMT genes play important roles in temperature-sensitive male sterile wheat

Temperature and drought are the major abiotic stresses during the reproductive stage that threaten the growth and development of wheat. In particular, male reproductive organs are known to be more sensitive to temperature stress than female organs in higher plants [55, 71]. Temperature stress may lead to complete sterility during reproductive development [54]. The two-line system, which takes advantage of photoperiod and/or temperature-sensitive genic male sterile (P/TGMS) lines, plays a great role in hybrid seed production of wheat [51]. In the TGMS line of wheat, the pollen viability is dramatically decreased to complete sterility at a certain low temperature occurring during reproductive development [52, 53]. Recent studies have shown that DMT genes contribute to plant stress responses and development by de novo establishment and subsequent maintenance of DNA methylation [72]. However, the transcript abundance of DMT genes in temperature-sensitive genic male sterile wheat has rarely been studied.

In this study, we investigated the transcript abundance of the TaDMT genes in the temperature-sensitive genic male sterile line BS366 based on the published GeneChip® data under sterile and fertile conditions. The results showed that there were 13 TaDMT genes that were expressed, and 10 of them were differentially expressed in BS366 under sterile and fertile conditions. Almost all the differentially expressed TaDMT genes were upregulated, and nearly all differential expression of TaDMT genes occurred during the 2.2-, 2.5- or 3.0-mm anther stage (Fig. 4b). Therefore, we speculated that the 2.2-mm, 2.5-mm and 3.0-mm periods are the key periods affecting male sterility in BS366. We further analyzed and compared the expression levels of the 9 DMT genes in three fertile wheat lines and their different fertility-restored hybrids. We found upregulated expression was detected for *TaCMT-D2*, *TaCMT-B5*, *TaMET1-B1*, *TaMET1-B2*, *TaMET1-B3*, *TaDRM-U6*, *TaDRM-B7*, and *TaDRM-D9* in the low fertility-restored hybrid GLDS×BS366, while their expression was downregulated or did not differ in TY806 × BS366 (high fertility-restored hybrid) and MC159 × BS366 (medium fertility-restored hybrid) under fertile and sterile conditions. Furthermore, the transcript levels of *TaCMT-D2*, *TaCMT-B5*, *TaMET1-B1*, *TaMET1-B2*, *TaMET1-B3*, *TaDRM-U6*, *TaDRM-B7*, and *TaDRM-D9* presented heterotic expression in GLDS×BS366, but not in other two cross combinations (Fig. 5). Two explanations were presented which might help to decipher this result: 1) GLDS×BS366 is the least fertile combination among these three cross combinations, and the expression patterns of these genes might be correlated with fertility in the three hybrids; 2) the heterotic expression in TY806 × BS366 and MC159 × BS366 might be repressed due to the paternal imprinting effect. Then, the tissue-specific expression patterns of the eight TaDMT genes were conducted using another three different fertility-restored hybrids. After analysis of the transcript abundance of TaDMT genes step-by-step under sterile and fertile conditions, we concluded that *TaCMT-D2*, *TaMET1-B1*, and *TaDRM-U6* were the candidate TaDMT genes that might participate in male sterility in BS366.

To search target genes of the three candidate TaDMT genes, correlation analysis of the expression levels among the DEGs in BS366 under fertile and sterile conditions was conducted. We found that *TaRAFTIN1A*, an important gene for pollen development, was highly negatively correlated with *TaMET1-B1* (r = − 0.99). To verify the relationship between *TaMET1-B1* and *TaRAFTIN1A*, the transcript and DNA methylation levels of *TaRAFTIN1A* was further examined in this study. We found that the expression levels of *TaRAFTIN1A* in BS366 and its three hybrids were significantly downregulated under sterile conditions than that under fertile conditions (Fig. 9a) and the average CG and CHG methylation levels in BS366 were with a difference by more than 20% under sterile conditions than that under fertile conditions (Fig. 9b), but not in the fertile line 07GF83. From the results, we further speculated that the interaction between *TaMET1-B1* and *TaRAFTIN1A* might play an important role in the male sterility of BS366. *TaMET1-A1*, *TaMET1-B1*, and *TaMET1-D1* are the three wheat homoeologs of *AtMET1* in *Arabidopsis* and *OsMET1B* in rice. Previous studies have shown that only the loss-of-function mutation of *ATMET1* resulted in almost all loss of CG methylation in *Arabidopsis thaliana* [13, 14]. Likewise, in rice, a null mutation of *OsMET1B* led to genome-wide hypomethylation [16]; however, a knockout mutant of *OsMET1A* (*OsMET1–1*) did not show alteration of DNA methylation at the assessed marker loci [17]. These results suggest the importance of *TaMET1-A1*, *TaMET1-B1*, and *TaMET1-D1* in the maintenance of CG methylation in wheat. In this study, *TaMET1-A1* was not expressed in BS366. Additionally, *TaMET1-B1* was upregulated, while *TaMET1-D1* was downregulated in BS366 under sterile conditions compared to fertile conditions. This phenomenon has also been found in the MIKC-type MADS-box genes [49]. The number of balanced transcript abundance from all three homoeologs of MIKC-type MADS-box gene triads was relatively low compared with an average assessment of all wheat genes [49]. The unbalanced homoeolog expression pattern might predict different functions of different homoeolog alleles.

## Conclusions

In this study, DNA methyltransferase (DMT) genes in wheat (*Triticum aestivum* L.) based on genome-wide levels were identified and transcriptionally characterized to assess the regulation of DMT genes in the fertility of the temperature-sensitive genic male sterile (TGMS) wheat line BS366. We presented a detailed phylogenic overview of wheat DMT (TaDMT) genes. Homoeolog retention for TaDMT genes was significantly above the average retention rate for whole-wheat genes. We also found extensive expansion of the TaDRM subfamily, and tandem duplications might play a crucial role in the expansion of the TaDRM subfamily. Following transcriptional analysis of TaDMT genes in the TGMS line BS366 and its hybrids with six other fertile lines under sterile and fertile conditions, we concluded that *TaCMT-D2*, *TaMET1-B1*, and *TaDRM-U6* might be involved in the male sterility of BS366. Moreover, a correlation analysis showed that *TaMET1-B1* might negatively regulate the expression of *TaRAFTIN1A*, an important gene for pollen development. Our findings could provide novel insights into the effects of the DMT genes on TGMS wheat.

## Methods

### Genome-wide identification of DMT genes

Functional annotations of wheat, rice, and *Arabidopsis* genes were downloaded from the Ensembl (http://plants.ensembl.org/info/website/ftp/index.html). A hidden Markov model (HMM) analysis was performed in the Protein family database (Pfam) to extract HMM profiles for the DNA methylase domain (PF00145), Chromo domain (PF00385), BAH domain (PF01426), and DNMT1-RFD domain (PF12047) from all species. The software hmmer was used to search for the DMT genes (E < 1e-10). Subsequently, Pfam and Conserved Domain Database (CDD, https://www.ncbi.nlm.nih.gov/cdd/) were used to confirm all the DMT genes containing the conserved domain. A total of 75 sequences were identified in the wheat genome. After excluding splice variants and keeping only the first variant, 52 genes were used for further analysis.

### Maximum likelihood phylogeny, conserved motif, gene structures, and *cis*-acting elements of DMT genes

Related DMT sequences from wheat, rice, and *Arabidopsis* were aligned by Clustal W, and then a phylogenetic tree was inferred using MEGA X [60] software by the maximum likelihood method (bootstrap value = 1000). The motifs of DMT genes in wheat were analyzed with the MEME database (http://meme-suite.org/tools/meme), Parameter settings were -nmotifs 10, and the remaining parameters were the default. Based on genetic feature format data from the wheat genome, the gene structures of DMT genes were analyzed using Tbtools software [73]. To analyze the *cis*-acting elements of *TaCMT-D2*, *TaMET1-B1*, *TaDRM-U6*, and *TaRAFTIN1A*, the upstream sequences (2000 bp) of the start codon were retrieved from the wheat genome database, and then the *cis*-acting elements were analyzed by using the PlantCARE tool [74].

### Naming of DMT genes in wheat

Naming wheat DMT genes, genome location and phylogenetic relationships were considered. The name of each gene started with Ta, an abbreviation for *Triticum aestivum*, and was followed by the name of the Arabidopsis gene from this subfamily. The subsequent name was A, B or D, indicating the subgenome that the DMTs were located in. Homoeologs were identified by phylogeny. Putative homoeologs have identical gene names except for the subgenome identifier (e.g., *TaMET1-A1*, *TaMET1-B1*, and *TaMET1-D1*). Genes belonging to the same subfamily but different triads within the same subgenome were consecutively numbered (e.g., *TaCMT-A1* and *TaCMT*-A2). Paralogs were distinguished by consecutive numbers separated by a dash (e.g., *TaDRM-A1–1* and *DRM-A1–*2).

### Plant materials, RNA isolation, and RT-PCR

Wheat (*Triticum aestivum* L.) cv. BS366, J411, GLDS, TY806, MC159, and hybrids GLDS×BS366, MC159 × BS366, and TY806 × BS366, 07YH91–43 × BS366, 07GF83 × BS366, and 7P395 × BS366 were used for reverse transcription-polymerase chain reaction (RT-PCR) analysis. Wheat TGMS line BS366 (Beijing Sterility 366) was selected from a natural mutant of doubled haploid lines (offspring of Jingnong8121/E8075–7) in the experimental fields in Beijing (China, N 39°54′, E 116°18′). BS366, GLDS, TY806, MC159, 07YH91–43, 07GF83, and 7P395 were bred in the Beijing Engineering and Technique Research Center for Hybrid Wheat. J411 (Jing411) was bred by Beijing Seed Company and was the offspring of Fengkang2/Changfeng 1. All the materials used in the study have the permissions for scientific research and publication. The plants were grown in the soil of plastic pots embedded in the ground according to Tang et al. [52] and they were vernalized naturally in the field. After the tip of the fourth leaf had emerged, the plants were moved to the fertility conditions, 20 °C with a 12/12-h light/dark cycle in phytotrons (Koito, Tokyo, Japan) for the entire experimental period, except during low-temperature treatment. When the tip of the flag leaf had half-emerged, part of the plants were treated for the sterility treatment, 10 °C with a 12/12-h light/dark cycle for 5 days. The anthers of J411, BS366, GLDS, TY806, MC159 GLDS×BS366, MC159 × BS366, and TY806 × BS366 and the roots, stems, leaves, and anthers of 07YH91–43 × BS366, 07GF83 × BS366, and 7P395 × BS366 were then collected and snap-frozen at − 80 °C. Total RNA was extracted using TRIzol reagent (Invitrogen, Carlsbad, CA, USA) according to the manufacturer’s instructions. First-strand cDNA was synthesized using a Takara PrimeScript™ RT Reagent Kit (Takara Biotechnology [Dalian] Co., Ltd., Dalian, China) using a random primer. An Eco Real-Time PCR System (Illumina, San Diego, CA, USA) with Takara SYBR® Premix Ex Taq™ (Tli RNase H Plus; Takara Biotechnology [Dalian] Co., Ltd.) was used for RT-PCR. *Actin* acted as an internal control for the wheat expression studies. Primers were designed using Premier 3.0 (v. 0.4.0) (Supplemental Table S2). Relative gene expression levels were calculated using the 2^−ΔΔCT^ method [75].

### Expression analysis of DMT genes using GeneChip®

GeneChip® data were from Tang et al [52]. Five developmental stages categorized by anther length (1.5-mm, 2.0-mm, 2.2-mm, 2.5-mm, and 3.0-mm) refer to 10 time points from BS366, a wheat TGMS line, under fertile and sterile conditions. Probe sets were considered differentially expressed with statistical significance when *P*-values were 0.05, corresponding to the false discovery rate of 5%. Gene annotations were obtained by Batch BLASTx (E-value < e-10) using the sequences of Affymetrix probe sets [52]. Hierarchical clusters of all annotated genes and the expressed TaDMT genes were generated according to their relative transcript levels in 10 samples using the R package *pheatmap*. The R package *topGO* were used to cluster the functional categories across the list of significantly enriched functional GO terms.

### Pollen iodine staining of wheat lines

Over 240 anthers from each wheat line were used for pollen iodine staining, and pollen iodine staining was performed according to [61]: the mature anther was squeezed using small tweezers to release the pollen grains; then, 1 to 2 drops of I2-KI solution [6.7‰ (g/ml) KI and 3.3‰ (g/ml) I2] were added. Finally, the images were obtained using an Olympus BX41 laboratory microscope (Olympus, Tokyo, Japan).

### Bisulfite sequencing

Genomic DNA for bisulfite sequencing was extracted during meiosis from the anthers of BS366 and 07GF83 plants under fertile and sterile conditions. The bisulfite modification of genomic DNA (~ 2 μg) from 8 samples was performed with sodium bisulfite using an EZ DNA Methylation-GoldTM Kit (Zymo Research, Orange, CA, USA). The bisulfite conversion step was extended by the addition of the denaturation step (98 °C for 10 min) and 2.5-h incubation at 64 °C. The CpG island was predicted by EMBL-EBI database (https://www.ebi.ac.uk/) and primers for the target site were designed using PyroMark Assay Design 2.0 (Qiagen). The primers of *TaRAFTIN1a* were shown in Supplemental Table 2. PCR reaction was conducted with Taq Polymerase (Shanghai Sangon Biotech Corp., Shanghai, China) using an ABI Veriti™ 96-Well Thermal Cycler (Applied Biosystems, Foster City, CA, USA). The PCR products were cloned into the pUC18-T vector (Shanghai Sangon Biotech Corp.). Ten clones per amplicon were selected for sequencing. The results were aligned by Clustal X 2.1 (http://www.clustal.org/clustal2/) and analyzed with CyMATE (http://www.cymate.net/). The methylation levels which were expressed as the percentage (%) for each of the three types of cytosines, CG, CHG, and CHH, were calculated by dividing the number of converted (methylated) cytosines by the total number of cytosines within the assay.

## Supplementary Information

**Additional file 1: Table S1.** List of all DMT genes in wheat. **Table S2.** Primer information used in this study.

**Additional file 2: Fig. S1.** Conserved domain analysis of DMT genes in wheat. **Fig. S2.** Conserved motifs of DMT genes in wheat. **Fig. S3.** Analysis of the *cis*-acting elements in the promoters of *TaCMT-D2*, *TaMET1-B1*, *TaDRM-U6*, and *TaRAFTIN1A*.

## Data Availability

The microarray data used for this study are deposited at the National Center for Biotechnology Information Gene Expression Omnibus (https://www.ncbi.nlm.nih.gov/geo/) under accession number GSE171305.

## Abbreviations

DMT: DNA methyltransferase
P/TGMS: Photoperiod and/or temperature-sensitive genic male sterile
MET1: Methyltransferase 1
CMT: Chromomethylase
DRM: Domains Rearranged Methyltransferase
DNMT2: DNA methyltransferase 2
RdDM: RNA-directed DNA methylation
siRNAs: Small interfering RNAs
DEGs: Differently expressed genes
GO: Gene Ontology
TEs: Transposable elements
BAH: Bromo-adjacent homology
