# Supplementary material for: Genome-wide identification and transcriptional characterization of DNA methyltransferases conferring temperature-sensitive male sterility in wheat
Source: BMC Genomics. 2021 Apr 29;22:310. doi: 10.1186/s12864-021-07600-7 (PMC8082647; doi:10.1186/s12864-021-07600-7)
Supplement: Supplementary file 2 — Additional file 2: Fig. S1. Conserved domain analysis of DMT genes in wheat. Fig. S2. Conserved motifs of DMT genes in wheat. Fig. S3. Analysis of the cis-acting elements in the promoters of TaCMT-D2, TaMET1-B1, TaDRM-U6, and TaRAFTIN1A. [file 12864_2021_7600_MOESM2_ESM.docx]

**Supplemental Figures and Legends**

**
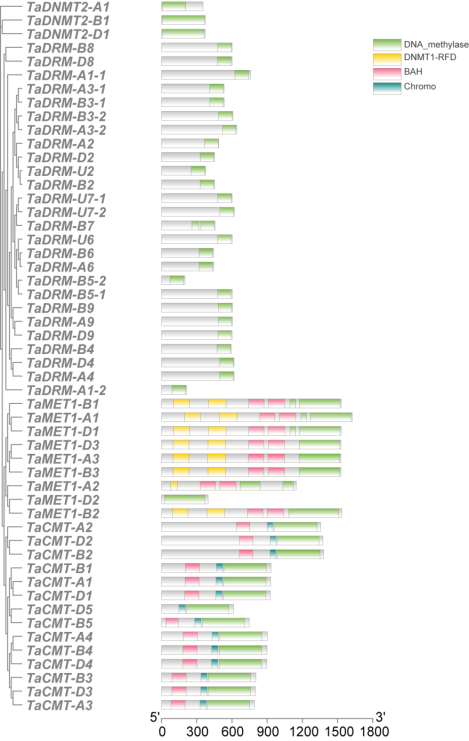
**

**Fig. S1.** Conserved domain analysis of DMT genes in wheat


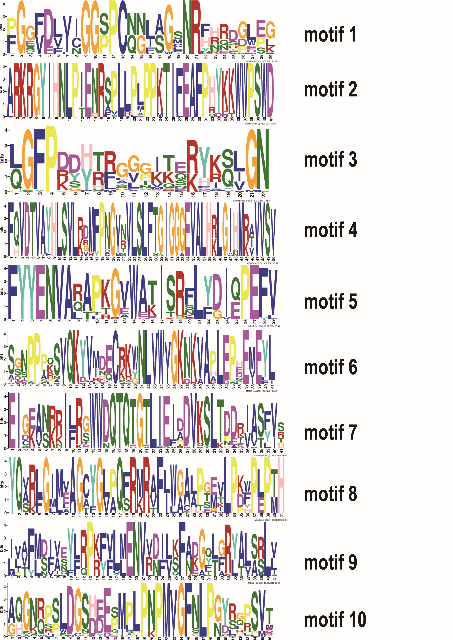


**Fig. S2.** Conserved motifs of DMT genes in wheat

**
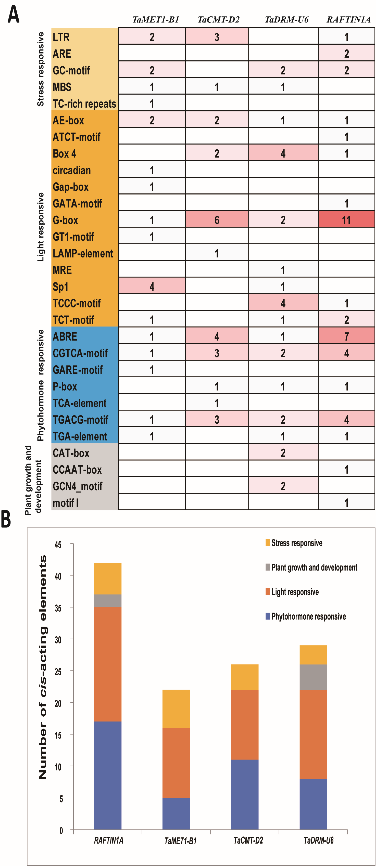
**

**Fig. S3.** Analysis of the *cis*-acting elements in the promoters of *TaCMT-D2*, *TaMET1-B1*, *TaDRM-U6*, and *TaRAFTIN1A*

**a** The numbers of different cis-acting regulatory elements in the promoters of *TaCMT-D2*, *TaMET1-B1*, *TaDRM-U6*, and *TaRAFTIN1A*. **b** The cis-acting elements in four categories were represented by different colors.
